# Molecular Mechanisms Underlying the Therapeutic Role of Vitamin E in Age-Related Macular Degeneration

**DOI:** 10.3389/fnins.2022.890021

**Published:** 2022-05-04

**Authors:** Genea Edwards, Caroline G. Olson, Carlyn P. Euritt, Peter Koulen

**Affiliations:** Department of Ophthalmology, Vision Research Center, School of Medicine, University of Missouri-Kansas City, Kansas City, MO, United States

**Keywords:** age-related macular degeneration (AMD), antioxidant, retina, tocopherol, tocotrienol, vitamin E

## Abstract

The eye is particularly susceptible to oxidative stress and disruption of the delicate balance between oxygen-derived free radicals and antioxidants leading to many degenerative diseases. Attention has been called to all isoforms of vitamin E, with α-tocopherol being the most common form. Though similar in structure, each is diverse in antioxidant activity. Preclinical reports highlight vitamin E’s influence on cell physiology and survival through several signaling pathways by activating kinases and transcription factors relevant for uptake, transport, metabolism, and cellular action to promote neuroprotective effects. In the clinical setting, population-based studies on vitamin E supplementation have been inconsistent at times and follow-up studies are needed. Nonetheless, vitamin E’s health benefits outweigh the controversies. The goal of this review is to recognize the importance of vitamin E’s role in guarding against gradual central vision loss observed in age-related macular degeneration (AMD). The therapeutic role and molecular mechanisms of vitamin E’s function in the retina, clinical implications, and possible toxicity are collectively described in the present review.

## Introduction

Nutrition has a significant influence on ocular health. Certain vitamins may prevent or slow the risk of several eye diseases like age-related macular degeneration (AMD), glaucoma, diabetic retinopathy (DR), and cataracts. Supplementation is oftentimes beneficial and necessary if dietary intake is below recommended guidelines. Compared to other organs of the body, the eye is particularly susceptible to oxidative stress. Finding a balance between free oxygen radicals and antioxidant vitamins E, A, and C may lower the threat of retinopathy. Of the fat-soluble vitamins, vitamin E is a powerful antioxidant occurring organically in foods that protect the body from free radicals that damage cellular processes. Vitamin E exists naturally in eight chemical forms: α (alpha), β (beta), γ (gamma) and δ (delta)-tocopherol and α (alpha), β (beta), γ (gamma) and δ (delta)-tocotrienol. Of all the forms, γ-tocopherol is the most common form found in a Western diet of plant oils, though α-tocopherol [D-α-tocopherol (RRR-α-tocopherol) configuration] is the most biologically active. Tocopherols are subject to oxidation, hence tocopheryl acetate (DL-α-tocopheryl acetate), tocopheryl succinate (DL-α-tocopheryl succinate), and tocopheryl nicotinate (DL-α-tocopheryl nicotinate)were created for their stability under oxidative conditions by converting the phenol group of the vitamin to an ester while tocopheryl nicotinate is esterified to a niacin (vitamin B3) molecule.

Tocotrienols are the less understood and considerably less widespread form of vitamin E. Tocotrienols differ in their side chains by containing three trans double bonds, making them much more flexible, putting greater stress on phospholipid membranes. They are found in cereal grains like barley, rice, and wheat. Palm oil is a great source of antioxidants that contains a significant amount of tocotrienols. Half of the natural vitamin E family are represented by tocotrienols, yet there are major gaps in the literature regarding the non-α-tocopherol forms. The biological actions of the differing homologs of vitamin E are diverse though they are structurally similar. The unsaturated side chains of tocotrienols appear to allow for more efficient tissue penetration and distribution (Sen et al., 2006). Research into tocotrienols has gained momentum in the last several decades and has changed the trend in research of vitamin E.

The focus of this review is to shed light on the molecular mechanisms involved in vitamin E signaling pathways as a potential therapy for AMD, a progressive and irreversible worsening of central vision, using a search of peer-reviewed articles in the PubMed^®^ database for biomedical literature focusing on the key words of the present review article and covering the literature published prior to submission of the present review article. Current treatments such as anti-angiogenic drugs or laser therapy slow down the progression but there is no cure. Results from population-based studies report no improvement with vitamin E supplementation (Taylor et al., 2002) while others tout the benefits for intermediate or advanced AMD (Lindblad et al., 1999; Chew et al., 2012). A synopsis of important preclinical and clinical studies involving vitamin E’s benefit toward preserving vision are presented here with the optimism that it will attract more research attention to its mechanism of action.

## Therapeutic Role of Vitamin E in Retinal Disease

Since its discovery a century ago (Evans and Bishop, 1922; Evans, 1925), vitamin E’s antioxidant, anti-inflammatory, and anti-apoptotic properties have made it a therapeutic option for neurodegenerative diseases. Its antioxidant properties were discovered in the 1930s from studies directed at animal fats (Olcott and Emerson, 1937). In addition to its ability to delay cellular injury, vitamin E also regulates inflammatory cytokines and cell-signaling mechanisms. Evidence indicating that the neurodegenerative process is associated with oxidative stress and inflammation has led to the idea that neurological conditions, especially retinal neurodegeneration may be prevented with vitamin E.

### Dietary Supplementation

Antioxidants take part in the crucial role of maintaining the health of retinal tissue, as the retina is highly susceptible to oxidative damage and free radicals. It naturally has a high amount of blood supply with extensive oxidative metabolism which can lead to increased amounts of free radical production and lipid peroxidation (Muller, 1992; Winkler et al., 1999). The therapeutic role of vitamin E in retinal disease pathogenesis has been widely explored yet the focus of the data has been on clinical studies in limiting the progression of retinal disease with vitamin E treatment, especially dry AMD. The molecular mechanisms of its therapeutic activity on degenerative retinal diseases like AMD remain lesser-known. Vitamin E’s importance, alone or in combination with additional vitamins such as vitamins A and C has been shown to maintain retinal structure and function. The most prevalent form of synthetic vitamin E is tocopheryl acetate, found commonly in supplements, especially widely popular ocular supplements containing the Age-Related Eye Disease Study (AREDS) and AREDS2 formulation which introduced zeaxanthin and lutein into the supplement (Lindblad et al., 1999; Chew et al., 2012). These studies are important clinical trials funded by the National Eye Institute and considered to be the gold standard in promoting eye health. These supplements have been clinically shown to slow the progression of advanced AMD (Snodderly, 1995). Additional intake of lutein, zeaxanthin, and other carotenoids with the reduction in zinc and the absence of beta-carotene from the original AREDS formulation has shown to be beneficial alongside vitamin E (Table 1; Cho et al., 2008; Abdel-Aal el et al., 2013; Yang S. F. et al., 2016). A recent comparative study looked at the concentrations of vitamin E in national name brand vitamin supplements recommended for patients at risk for macular degeneration and concluded that levels were slightly higher than the label indicated, but not enough to cause systemic toxicity (Fleissig et al., 2021).

**TABLE 1:** Comparison of AMD nutritional supplements.

Commercially available formulas									
Nutrient	AREDS*	AREDS2	B&L Preservision AREDS	B&L Preservision AREDS2	B&L Ocuvite Eye Health	Alcon Systane I-Caps AREDS	Alcon Systane I-Caps AREDS 2	Biosyntrx Eye and Body Complete	Eye Science Macular Health Formula
Vitamin C	400 mg	400 mg	226 mg (ascorbic acid)	250 mg (ascorbic acid)	150 mg (ascorbic acid)	2226 mg (ascorbic acid)	250 mg (ascorbic acid)	251 mg (ascorbic acid and ascorbyl palmitate)	500 mg (ascorbic acid)
Vitamin E	400 IU	400 IU	90 mg (dl-alpha tocopherol acetate)	90 mg (dl-alpha tocopherol acetate)	20 mg (d-alpha tocopherol)	120 IU (dl-alpha tocopheryl acetate)/80 IU (d-alpha -tocopheryl acetate)	200 IU (d-alpha tocopherol acetate)	15 IU (d-alpha tocopheryl succinate and mixed tocopherols), 15 mg mixed tocotrienols	400 IU (d-alpha tocopheryl succinate)
Beta-carotene*	15 mg	–	4296 mcg	–	–	14320 IU	–	1000 IU (retinyl palmitate)	–
Copper (cupric oxide)**	2 mg	2 mg	0.8 mg (cupric oxide)	1 mg (cupric oxide)	1 mg (copper oxide)	0.8 mg (cupric oxide)	1 mg (cupric gluconate)	0.25 mg (copper sebacate)	2 mg (copper gluconate)
Lutein	–	10 mg	–	5 mg (marigold flower extract)	5 mg (marigold flower extract)	–	5 mg	10 mg	10 mg
Zeaxanthin	–	2 mg	–	1 mg (marigold flower extract or paprika fruit extract)	1 mg (marigold flower extract)	–	1 mg	3.13 mg zeaxanthin isomers, 2.13 mg zeaxanthin 3R, 3′R	2 mg
Zinc	80 mg	80 mg	34.8 mg (zinc oxide)	40 mg (zinc oxide)	9 mg (zinc oxide)	34.8 mg (zinc oxide)	12.5 mg (zinc oxide)	12.5 mg (zinc monomethionine)	40 mg (zinc oxide)
Omega-3 fatty acids	–	–	–	–	250 mg (160 mg EPA, 90 mg DHA)	–	–	–	–

*Not recommended for smokers. **Added to avoid zinc-related copper deficiency. Formulas are based on the NEI-funded Age-Related Eye Diseases Studies (AREDS and AREDS2). Supplements that are made from entirely natural sources contain d-alpha-tocopherol. This also is referred to as RRR-alpha-tocopherol. D-alpha-tocopherol is the most bioavailable form of alpha-tocopherol, meaning it’s the type that is preferred for use by your body and is better absorbed and utilized than other forms. Dl-alpha-tocopherol is a synthetic form of alpha-tocopherol. This synthetic form of alpha-tocopherol is less bioavailable than the d-alpha-tocopherol and is only half as potent, notes the Oregon State University, Linus Pauling Institute. This form of alpha-tocopherol is frequently found in nutritional supplements and fortified foods. Conversion: 1 mg of alpha-tocopherol is equivalent to 1.49 international units (IU) of the natural form (d-alpha tocopherol) or 2.22 IU of the synthetic form (dl-alpha tocopherol), 1 mg of beta carotene equals 1667 IU.

Dietary supplementation with antioxidants can inhibit complications of diabetes due to oxidative stress and abnormal ATPase activity in the retina (Kowluru et al., 1996, 1999, 2001). Retinal blood flow improvement in patients with diabetes as evidenced by diabetes-induced electroretinogram (ERG) and retinal vascular permeability (RVP) abnormalities have been seen with vitamin E supplementation (Kunisaki et al., 1995, 1998; Timothy et al., 2004). Other serious retinal diseases and injuries of importance in which vitamin E has been shown to also provide a protective effect include photic injury, oxidative injury, retinal edema, uveitis-associated macular edema, and glaucomatous damage (Tanito et al., 2002; Ohira et al., 2003; Aydemir et al., 2004a,b; Nussenblatt et al., 2006; Engin et al., 2007; Zapata et al., 2008).

### Macular Degeneration and Vitamin E

The highest concentrations of vitamin E are found inside the retinal pigment epithelium (RPE) followed by the outer segments of the photoreceptor cells (Stephens et al., 1988). Photoreceptor cell death (Dunaief et al., 2002), lipofuscin accumulation (Dorey et al., 1989; Finnemann et al., 2002), and metabolic dysfunction of RPE are the main contributing factors in AMD (Brown et al., 2019). Oxidative stress is known to play a part in photoreceptor cell death and overall damage in many retinal diseases including retinitis pigmentosa (RP), diabetic retinopathy (DR), AMD, and glaucoma. Research studies report that macular degeneration developed in monkeys after a diet deficient in vitamin E. Lesions were characterized by large, focal disruption of photoreceptor outer rod segments (Hayes, 1974b). Indication of mitochondrial oxidative stress in RPE linked metabolic dysfunction between photoreceptors and RPE suggesting a possible mechanism for AMD in superoxide dismutase 2 (Sod2)-KO mice (Brown et al., 2019). Photoreceptor outer segment degeneration was reported in rats that were given a diet lacking vitamin E due to enhanced activity of lysosomal enzymes in the RPE (Amemiya, 1981). These studies call attention to the consequences of a diet deficient in vitamin E to guard against oxidative stress induced by lipid peroxidation and suggest vitamin E’s role as a treatment strategy in preserving retinal function in AMD.

Scientific literature has also identified an inflammatory role in AMD (Ambati et al., 2013; Kauppinen et al., 2016). Inflammatory cytokines, complement system, macrophage involvement, and more recently, inflammasomes of the innate immune system have been shown to be involved in the pathogenesis of AMD (Liu et al., 2011; Chen and Smith, 2012; Shin and Bayry, 2013; Knickelbein et al., 2015; Yang Y. et al., 2016). Oxidative stress coupled with inflammation performs a role in disease progression to the intermediate state characterized by accumulation of drusen, lipofuscin deposits that build up in the Bruch’s membrane. Constituents of drusen such as amyloid-β,7-ketocholesterol, carboxyethylpyrrole protein (CEP)-adducts, and advanced glycation end products (AGE)-adducts may elicit local complement activation (Crabb et al., 2002; Dentchev et al., 2003; Glenn and Stitt, 2009; Rodríguez and Larrayoz, 2010). Of those with early to intermediate AMD, 15–20% will develop into late-stage AMD (Sunness et al., 1997). Evidence of increased levels a marker of inflammation, high-sensitivity C-reactive protein (hsCRP), may predict the risk of macular degeneration. A study from 2013 looked at hsCRP in blood samples of men and women and observed a significantly increased risk of AMD for high versus low hsCRP levels (Mitta et al., 2013). CRP potentially mediates complement activation and may have significant roles in therapeutic intervention in AMD. Activation of complement by CRP was demonstrated with exogenous addition of CRP by the formation of complement component iC3b in A2E-laden RPE cells bathed in normal human serum (Zhou et al., 2009). The fluorophore molecule A2E (N-retinylidene-N-retinylethanolamine) is short for two all-trans-retinal molecules (vitamin A aldehyde) and one ethanolamine molecule (Lamb and Simon, 2004). In Sparrow et al. (2012), they showed that pre-treatment with 100 μM of vitamin E for 24-h suppressed complement activation evident by reduction of iC3b production in mature retinal pigment epithelium-19 (ARPE-19) cells with A2E accumulation (Sparrow et al., 2012).

Tocotrienols have been shown to inhibit angiogenesis, the development of new capillaries from established blood vessel networks (Miyazawa et al., 2008). Excessive and abnormal growth of new blood vessels frequently occurs in neovascular or “wet” AMD. Research performed with human umbilical vein endothelial cells (HUVECs) by Miyazawa et al., 2008 concluded that tocotrienols halted proliferation induced by growth factors, cellular migration, and tube formation. Tocotrienols also displayed suppression of tumor cell-induced angiogenesis in mouse dorsal air sac (DOS) assay, tocopherols did not (Miyazawa et al., 2008). Evidently, the differences in the biological activity of the two forms of vitamin E are not redundant and the different isoforms of vitamin E should be individually considered.

## Vitamin E Deficiency

Our bodies need regular consumption and supply of vitamin E stores. In 2000, the Food and Nutrition Board of the Institute of Medicine recommended a 15 mg typical daily allowance of vitamin E for adults (Medicine, 2000). Vitamin E is lipid soluble, so any deficiencies are likely caused by dietary fat absorption or metabolism.

### Symptoms

Deficiency in vitamin E leads to characteristic, irreversible changes in retinal structure and function. Described changes include progressive neurological syndrome, pigmentary retinopathy, cerebellar ataxia, loss of position and vibration sense, pes cavus, scoliosis, and generalized muscle weakness (Hayton et al., 2003). Factors leading to deficiency in vitamin E can include environmental or nutritional influences, genetic entities, iatrogenic, or experimentally induced sources. Mutations found in α-tocopherol transfer protein (α-TTP) lead to ataxia, a decline in the coordination of voluntary muscle movements, with isolated vitamin E deficits (Ouahchi et al., 1995). Vitamin E deficiencies are rare, but cases have been seen in children diagnosed with abetalipoproteinemia and familial hypobetalipoproteinaemia. These syndromes are lipoprotein deficiency disorders causing a large amount of fat to build up in the blood due to a lack of a protein that breaks down the fat molecules (Lloyd, 1973). A very small study of children diagnosed with chronic cholestasis and low blood serum vitamin E and A concentrations all developed abnormal flash ERGs and half had abnormal visual evoked potentials (VEPs; Bishara et al., 1982). Patients with abetalipoproteinemia and severe vitamin E deficiency demonstrated abnormal visual electrophysiology (Bishara et al., 1982). Clinical retinal manifestations include the development of progressive pigmentary retinopathy and subnormal mixed cone-rod ERG amplitudes. Initial treatment with oral vitamins E and A is advised (Alvarez et al., 1983; Chowers et al., 2001). Cystic fibrosis and cholestatic liver disease are also implicated in vitamin E deficiency with ocular findings largely revealing a decrease of the ERG b-wave, abnormalities of eye movement, and retinal degenerative changes (Alvarez et al., 1983).

### Retinopathy of Prematurity

Infants, especially premature babies, have an increased susceptibility to oxidative damage due to their exposure to large amounts of free radicals during the birth process as their lungs adapt to their new environment as well as having vitamin deficiencies (Muller, 1992). This is exacerbated in premature babies with respiratory distress syndrome of the newborn, in which they are administered supportive oxygen in the hospital, causing further free radical formation (Muller, 1992). Premature infants can have vitamin E deficiency which can manifest as hemolytic anemia and impaired coordination. Retinopathy of prematurity (ROP) is often treated with vitamin E supplementation in order to scavenge free radicals created in the hypoxic birth process. In most newborns, vitamin E is derived from breastmilk and reaches normal levels after a few weeks. Vitamin E is easily found in a balanced diet, however, a diet high in processed foods or low in fat can lead to a deficiency in vitamin E. This was studied in lactating women and those with diets high in processed foods had lower vitamin E levels in their breastmilk (Amorim et al., 2021).

### Bioavailability

Absorption of vitamin E is controlled by transporters including the multidrug resistance protein 1 (MDR1) and ATP-binding cassette transporter B1 (ABCB1) expressed on the apical surface of enterocytes. Drug interactions can occur at this point in the process as certain medications and herbal supplements including St. John’s wort can alter expression of these transporters (Podszun and Frank, 2014). Delivery of vitamin E to its target tissues is a necessary process that involves a number of lipoproteins for transport of vitamin E’s hydrophobic characteristics, and binding proteins which allow for transport intracellular and extracellularly (Table 2). Although γ-tocopherol is the most abundant isoform, its bioavailability is limited. Studies demonstrated that concentrations of γ-tocopherol, but not α-tocopherol, declined dramatically in plasma and lipoproteins of normal individuals 24-h after ingestion. Moreover, studies in patients post-surgical for gall bladder procedures, revealed secretion of γ-tocopherol in bile is preferential, suggesting the liver distinguishes between α- and γ-tocopherol secretion (Traber and Kayden, 1989). The discrimination of γ- in favor of α-tocopherol is due, in part, to a higher affinity for transfer protein. The mechanism of transport that is specific to α-tocopherol occurs in the liver by a 32 kDa protein, α-tocopherol transfer protein (α-TTP), that facilitates its secretion from hepatocytes to extrahepatic tissues (Thakur et al., 2010). α-TTP was first reported in rat liver by Catignani and Bieri (1977). Originally thought to only be present in the liver, it is now widely accepted to be found in the brain, kidney, lung, and spleen (Hosomi et al., 1998; Copp et al., 1999; Yamaoka et al., 2008; Tamura et al., 2020). Additionally, it has also been found to be localized to human placenta and mouse uterus (Kaempf-Rotzoll et al., 2002, 2003; Rotzoll et al., 2008). Distribution of vitamin E intracellularly has been identified to be controlled by a novel, cytosolic 46-kDa α-tocopherol associated protein (α-TAP), which binds α-tocopherol by chylomicron formation and lipids in the liver (Stocker et al., 1999; Zimmer et al., 2000) and acts as a metabolizing enzyme by increasing the uptake and absorption of vitamin E and hence facilitates an anti-proliferative effect most notably in prostate cancer and as a tumor suppressor in cancer through a non-vitamin E mechanism. The highest amounts of the human homolog hTAP have been found in the liver, brain, prostate, and breast epithelial cells (Upadhyay and Misra, 2009; Tam et al., 2013). α-TTP and α-TAP are expected to be found in the retina since this neural tissue and its circuitry is an extension of the brain and nervous system (London et al., 2013; De Groef and Cordeiro, 2018).

**TABLE 2:** Vitamin E binding and transport proteins.

**Vascular transport**
**Protein**	**Gene**	**Function**
Chylomicron/Apolipoprotein B-48	APOB	transport under normal physiological conditions
High density lipoprotein/Apolipoprotein AI	APOA1	transport under normal physiological conditions
Low density lipoprotein/Apolipoprotein B	APOB	transport under fasting conditions
Very low density lipoprotein/several apolipoproteins	APOB, APOC1, APOC2, APOE	transport under normal physiological conditions
Afamin	AFM	binds hydrophobic molecules and may be involved in the transport of vitamin E across the blood-brain barrier
**Intracellular binding proteins**
**Protein**	**Gene**	**Function**
Alpha-tocopherol transfer protein	TTPA (known as TPP1)	intracellular transport protein
Scavenger receptor class B type 1 (SR-B1)	SCARB1	transfers vitamin E into the cell
ATP-binding cassette transporter A1	ABCA1	excretes vitamin E out of the cell
SEC14-like protein 2 [known as alpha-tocopherol-associated protein (short names: TAP, hTAP), squalene transfer protein, supernatant protein factor (short name: SPF)]	SEC14L2 (synonyms:C22orf6, KIAA1186, KIAA1658)	associates with α-tocopherol by binding hydrophobic molecules for intracellular transport
SEC14-like protein 3 (aka Tocopherol-associated protein 2), SEC14-like protein 4 (aka Tocopherol-associated protein 3)	SEC14L3 (synonym:TAP2), SEC14L4 (synonym:TAP3)	not investigated thoroughly
Saposin B	PSAP	has specific binding site for γ tocopherol

### Transport Across Blood Barriers

Transport of vitamins and essential nutrients through the blood-retinal barrier (BRB) is facilitated by membrane permeability and regulation of tight junctions of the retinal capillary endothelial cells (inner BRB), analogous to the blood-brain barrier (BBB; Campbell and Humphries, 2012), and RPE cells (outer BRB). Inward and outward movement of fluid and molecules between blood and retina is restricted by these structures. DR and AMD are directly linked with alterations of the BRB (Cunha-Vaz et al., 2011). To circumvent the BRB, intravitreal injection of steroids and anti-vascular endothelial growth factor (VEGF) treatments have become more widely administered in recent years. Modulation of the inner BRB to enhance systemic therapeutic intervention may lead to better options in controlling retinal diseases (Campbell and Humphries, 2012). Aeschimann et al. (2017) has shown that vitamin E delivery can be effectively transported to tissues protected by an endothelial barrier utilizing HUVECs. α-TTP displays a tendency to aggregate into stable high molecular weight oligomers which then transport α-tocopherol across endothelial barriers but not through epithelial barriers.

Experimentally induced vitamin E deficiency is a way of evaluating the effects of decreased levels of vitamin E found in the retina. Interrelationships of vitamin E and A have been studied and was found that a diet fed to rats lacking in both vitamin E and A accelerated loss of photoreceptor cells but the amount of cell death varied according to the quantity of vitamin A provided in the diet (Robison et al., 1980). Dietary vitamin E must cross the BRB from the circulating blood to be effective in protecting the retina. Lipid transport by high-density lipoprotein (HDL) via scavenger receptor class B type I (SR-BI) in the retina has been identified in the transport process (Tachikawa et al., 2007). Tserentsoodol et al. (2006) showed an internal lipid transport mechanism that involves HDL-like particles and SR-BI proteins are found in retinal pigment epithelium/choriocapillaris (CC) regions, Müller cells, ganglion cells, and as well as primate photoreceptors. Experimental evidence using α-TTP null mice fed a diet deficient in vitamin E leads to a severe deficiency of vitamin E (a rare condition), enhances lipid peroxidation in the retina, and accelerates degenerative changes in the retina with age (Tanito et al., 2007).

### Animal Models of Retinal Vitamin E Deficiency

Rodent models of experimental vitamin E deficiency can provide clues to vitamin E’s role in disease processes of the retina (Elizabeth Rakoczy et al., 2006). Through disease models, we have learned that vitamin E and A maintain various structures of the retinal tissue, yet in their absence, results in lipofuscin deposits in the RPE and loss of rod nuclei in rats fed a diet lacking vitamin E and A (Robison et al., 1979, 1980). Studies using frog retinal outer rod segments revealed stabilization of membrane fluidity due to α-tocopherol (Moran et al., 1987). Alterations in membrane fluidity, lipid peroxidation, and irreversible loss of long-chain polyunsaturated fatty acids (LC-PUFAs) were also indicated in the rat (Goss-Sampson et al., 1998). Other models of experimentally induced vitamin E deficiency include monkey and bovine, which show similar alterations in loss of structural integrity to rod outer segment membranes (Hayes, 1974a; Farnsworth and Dratz, 1976; Guajardo et al., 1999). The symbiotic relationship between the structures of the outer BRB that include the RPE/Bruch’s membrane/choriocapillaris (CC) complex is lost in AMD and suggest vitamin E’s transport mechanism is ultimately compromised (Bhutto and Lutty, 2012; Hosoya and Kubo, 2014; Tisi et al., 2021).

## Molecular Mechanisms of Vitamin E Signaling in the Retina

**FIGURE 1:**
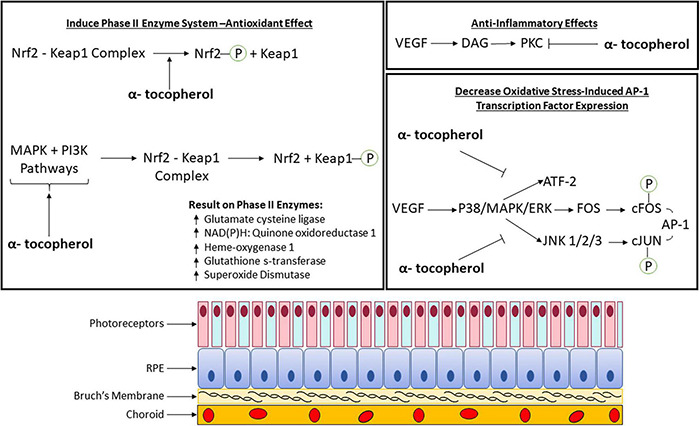
Molecular pathomechanism of vitamin E (α-tocopherol) with phase II enzyme system, anti-inflammatory, and oxidative stress involvement in the retina. AP-1, Activator protein-1; ATF-2, activating transcription factor 2; DAG, diacylglycerol; ERK, extracellular-signal-regulated kinase; FOS, proto-oncogene c-Fos; JNK, c-Jun N-terminal protein kinase; Keap1, kelch-like ECH-associated protein 1; MAPK, mitogen-activated protein kinase; Nrf2, nuclear factor erythroid 2-related factor 2; P, phosphate group; PI3K, phosphoinositide 3-kinase; PKC, protein kinase C; VEGF, vascular endothelial growth factor.

Vitamin E is known to activate kinases and transcription factors that regulate gene expression (Figure 1). Signaling pathways that are associated with the pathophysiology of macular degeneration such as the mitogen-activated protein kinase (MAPK) signaling pathway, which is stimulated by mitogens, hormones, growth factors, cytokines, oxidative stress (Kyosseva, 2016), and the transcription factor, nuclear factor erythroid 2-related factor 2 (Nrf2), regulates genes involved in the oxidative stress response (He et al., 2020). The most widely known signaling pathway associated with the retina and macular degeneration is the vascular endothelial growth factor (VEGF) signaling pathway (Kowanetz and Ferrara, 2006).

### Distribution of Vitamin E in Ocular Tissues

Much is still not understood on the signaling mechanism of vitamin E’s neuroprotective and cytoprotective effects in the retina. It has been shown that levels of α-tocopherol are higher in the retina than in the vitreous and choroid. These findings correlate with serum levels of α-tocopherol (Bhat, 1986). Results from a comparative study to determine distribution differences in rat eye tissue by administration of a 5 μL eye drop of either tocopherol or tocotrienol concluded that the α-tocotrienol concentration increased in all ocular tissues. Results showed that α-tocopherol did not increase significantly nor did γ-tocopherol and γ-tocotrienol differ significantly. Noteworthy increases in total vitamin E were found in the neural retina, eyecup, and crystalline lens (Tanito et al., 2004).

### Role of Vitamin E in Retinal Layers

Photoreceptors and especially the outer rod segments are the most vulnerable to oxidative damage through peroxidation because more than 65% of the membrane fatty acids are poly-unsaturated (Muller, 1992). Scavengers including glutathione peroxidase are upregulated in the photoreceptor outer segments in response to light exposure (Ohira et al., 2003). Levels of α-tocopherol in retinal cytosol also have a positive correlation with the antioxidant ability of vitamin A, suggesting a compounding effect (Guajardo et al., 1999). This part of the retina also sustains phototoxic damage due to intense light exposure over time which can be seen as lipofuscin granules (Muller, 1992; Winkler et al., 1999). Vitamin E can be beneficial to decreasing phototoxic damage because of its ability to decrease lipid peroxidation. Vitamin E deficiency accelerates RPE autofluorescent pigment deposition rates, possibly by enhancing the conversion of phagocytosed photoreceptor outer segment components into autofluorescent pigment granules (Muller, 1992; Winkler et al., 1999). This is likely a similar mechanism to age-related lipofuscin formation. Both are formed by autooxidation of photoreceptor disk membrane components.

The choroid and CC region can be affected by oxidative damage in AMD so there is potential for vitamin E to ameliorate these effects. As red blood cells pass through the CC region, hemoglobin precursors may undergo photoactivation (Winkler et al., 1999). Activating these precursors may generate reactive oxygen species which can then damage the RPE and Bruch’s membrane (Winkler et al., 1999). A deficiency of vitamin E in rats triggered an increase of lipofuscin content in melanocytes and fibroblasts of the choroid (Herrmann et al., 1984). These changes were not seen in the endothelial cells of the CC (Chapy et al., 2015).

### Interconnected Signaling Pathways

In addition to vitamin E’s oxygen scavenging properties, inhibition of cell growth and protein kinase C (PKC) activity has been observed (Saishin et al., 2003; Xu et al., 2004; Betti et al., 2006; Kim et al., 2010; Titchenell et al., 2012). It has also been shown to alter expression of transcription factors involved in gene expression (Zingg, 2015; He et al., 2020). More research is needed to demonstrate that vitamin E mediates signal transduction involved in macular degeneration pathology (Figure 1). A summary of these pathways as determined by published experimental studies is described in the sub-sections below.

### Models to Study Vitamin E Signaling

A common experimental model to study AMD and oxidative stress is the use of the ARPE-19 cell line, immortalized human RPE cells, and human telomerase reverse transcriptase (hTERT)-RPE. In Duncan et al., 2022 they demonstrate that α-tocopherol, γ-tocopherol, δ-tocopherol, and α-tocotrienol all exhibited similar, but not identical, antioxidant activity. In addition, exposure time is important for its protective properties against oxidative stress. Synthesis of new proteins was also found to be partially required with α-tocopherol, but not γ-tocopherol, within a 24-h period and before exposure to tertiary butyl hydroperoxide (tBHP) for optimal cytoprotection (Duncan et al., 2022). In another study, ARPE-19 cells were subjected to pre-treatment of ≥2.5 mM α-tocopherol, which significantly decreased oxidative stress-induced activator protein-1 (AP1) transcription factor expression at 14 h but was not further reduced with higher levels of α-tocopherol (Yin et al., 2011). The major AP1 transcription factor family of genes include JUN, FOS, and ATF, which are important regulators of redox, cellular homeostasis, and proliferation that can activate nuclear factor kappa-light-chain-enhancer of activated B cells (NFκB) and MAPK/extracellular-signal-regulated kinase (ERK) signaling pathways (Figure 1). Vitamin E pre-treatment also significantly improved viability in APRE-19 cells exposed to oxidative stress and quenched blue light induced lipofuscin autofluorescent pigment accumulations of A2E-epoxidation causing DNA damage and cell death in macular degeneration etiology, respectively (Sparrow et al., 2003; Kagan et al., 2012). It was also determined α -tocopherol, in combination with either zeaxanthin or lutein, provided better protection to A2E photooxidation than single antioxidant treatment (Kim et al., 2006).

Animal models are crucial for studying the mechanism of AMD pathogenesis and evaluating therapeutic options to prevent or slow disease progression. The features and stages of AMD are not all replicated in non-human primates due to the lack of a macula, but instead, horizontal visual streaks through the retina have similarities to the primate macula (Hughes, 1985). Researchers are still able to tease out valuable information on mechanistic and novel treatments. A table of commonly utilized models is listed in the following reviews (Pennesi et al., 2012; Fletcher et al., 2014). Vitamin E therapeutics can be administered to animals either systemically or topically to assess its effects on disease phenotypes. There is scope for further investigation here as this area is unexplored or currently under exploration during the writing of this review.

### Phase II Enzyme Inducer

Vitamin E has been shown experimentally to induce the phase II enzyme system. These enzymes are involved in drug metabolism in the liver and conjugate oxidized intermediates to form hydrophilic products that can be more easily excreted by the body. An upstream promoter regulatory element called the antioxidant-response element (ARE) regulates the expression of these enzymes. The ARE regulator is activated by Nrf2 transcription factors. A study using an acrolein (oxidant) model of AMD in human retinal pigment epithelium cells showed that α-tocopherol has been found to activate the nuclear factor erythroid 2-related factor 2 (Nrf2) pathway (Feng et al., 2010). This is done through cysteine residue oxidative modification within kelch-like ECH-associated protein 1 (Keap1) or phosphorylating Nrf2 (Feng et al., 2010). Activating this pathway upregulates phase II enzymes (Feng et al., 2010). Alternately, the same pathway can be activated by α-tocopherol by activating phosphoinositide 3-kinase (PI3K) and mitogen-activated protein kinase (MAPK) pathways which cause Keap1 phosphorylation (Figure 1; Feng et al., 2010). The importance of Nrf2 and upregulation of phase II genes has potential for neuroprotective application in AMD.

## Molecular Mechanisms of Vitamin E Signaling Outside of the Retina

Signal transduction pathways are modulated by vitamin E through several mechanisms relevant for its absorption, distribution, metabolism, and molecular functions. These include modulation of a variety of enzymes involved in signal transduction like cyclooxygenase-2 (COX-2), diacylglycerol kinase (DGK), 5-, 12-, and 15-lipoxygenases (LO), protein kinase B (PKB), PKC, protein tyrosine kinases (PTK), phospholipase A2 (PLA2), protein phosphatase 2A (PP2A), and protein tyrosine phosphatase (PTP) (Zingg, 2015, 2019).

### Modulation of Vitamin E

It is highly unlikely that one antioxidant is proven to be effective in absence of other members of a supporting team like vitamin C, selenium, vitamin A, CoQ10, and calcium to work efficiently (Golumbic and Mattill, 1941). Calcium plays a significant role in the metabolism of vitamin E. In studies of hepatocytes, calcium was shown to modulate vitamin E metabolism. Decreasing intracellular calcium levels led to a decrease in α-tocopherol levels (Pascoe and Reed, 1987).

### Peroxyl Radical-Scavenging System

Vitamin E’s antioxidant activity can scavenge reactive oxygen and nitrogen species. This can protect mono-unsaturated fatty acids (MUFAs/PUFAs) and their lipid mediators which are important for cellular functions (Zingg, 2019). The unusually high content of PUFAs in the membrane lipids are susceptible to oxygen damage when vitamin E is low. These fatty acids can play beneficial roles in preventing cancer, insulin resistance, non-alcoholic steatohepatitis (NASH), cardiovascular, and neurodegenerative disease (Zingg, 2019). It has been shown that mixed tocopherols as seen in a typical diet had more effect on several of the observed effects of tocopherol including decreasing lipid peroxidation, attenuating platelet aggregation, and decreasing arterial thrombosis than α-tocopherol alone (Liu et al., 2002). Modulation by vitamin E can also affect the stability and properties of the cell membrane, which can indirectly modulate the signaling properties of proteins in the membrane (Zingg, 2019). The different vitamin E analogs affect cellular signaling differently through signal transduction enzymes and influencing the translocation of receptors to the plasma membrane (Zingg, 2019). Supplementation with vitamin E succinate was shown to increase activity of glutathione reductase and thus increase glutathione concentrations (Rego et al., 1998). In addition, vitamin E has been demonstrated to affect oxidative actions related to stress. Stress-induced increase in lipid peroxidation caused by nitric oxide production is a process that can be decreased by vitamin E (Yargiçoğlu et al., 2003). Choroidal neovascularization, observed in “wet” AMD, is vulnerable to sub-retinal hemorrhages which may induce retinal degeneration by promoting lipid peroxidation from iron released from hemorrhages as oxyhemoglobin (HbO2) or methemoglobin (metHb). When porcine retinal homogenates were incubated with α-tocopherol or docosahexaenoic acid (DHA), a major fatty acid, α-tocopherol was more rapidly decomposed than DHA with metHb versus HbO2. α-tocopherol scavenged hemoglobin-induced lipid peroxyl radicals and was consumed in the process (Ito et al., 1995).

### Mitochondrial Dysfunction

Mitochondria are the primary user of oxygen for energy synthesis and reactive oxygen species (ROS) produced there can go to the cytosol, be neutralized by antioxidants, or remain within the mitochondria and interact with mitochondrial lipids, proteins, and DNA. These interactions can alter mitochondrial function by deactivating enzymes involved in the respiratory chain and citric acid cycle. Mitochondrial dysfunction has been implicated in diseases including aging, dementia, type 2 diabetes, and obesity (Napolitano et al., 2019). As the major antioxidant present in mitochondrial membranes, vitamin E can respond with peroxyl radicals and protect mitochondrial membranes from oxidative stress (Napolitano et al., 2019). This effect can also be seen from studies in allergic asthma. Interleukin-4 (IL-4) and 12/15 lipoxygenase (12/15-LOX) contribute to mitochondrial dysfunction in allergic asthma and can be reduced by vitamin E supplementation (Mabalirajan et al., 2009). The mechanism of IL-4 inhibition is thought to inhibit the binding of NF-κB and transcription factor Sp-1 with binding sites of the IL-4 promoter region (Mabalirajan et al., 2009). Interestingly, administration of vitamin E increased mice longevity by slowing mitochondrial degeneration (Mabalirajan et al., 2009).

### Vitamin E Signaling in Systemic Disease

In addition to Vitamin E’s antioxidant properties, vitamin E is known to have other beneficial effects in various disease processes including being anti-thrombotic, anti-neoplastic, anti-angiogenic, anti-inflammatory, and on levels of cholesterol (Pearce et al., 1992; Singh et al., 2005; Song and DeBose-Boyd, 2006; Zingg, 2019; Ziegler et al., 2020). Anti-proliferative and apoptotic properties have also been observed through studies examining its inhibitory effects on mouse mammary cells. This effect is thought to be due to its ability to reduce PKC activation and can be extrapolated to modulating general mammary gland development, function, and modification (McIntyre et al., 2000). Anti-neoplastic actions of tocopherol have been described in breast, colon, and prostate cancer cells (Betti et al., 2006). This is thought to be due to a decrease in PKC-α activity and decreased expression of cell cycle-related proteins (Betti et al., 2006). This PKC-α effect can also decrease activation of MAPK/ERK (Betti et al., 2006) and modulate gene expression, including certain proteins that control cell cycle progression, including cyclin D, cyclin E1, p27, and p53 (Betti et al., 2006). γ-tocopherol may have stronger anti-inflammatory and anti-neoplastic effects than α-tocopherol through increased inhibition of COX2 (Betti et al., 2006).

Vitamin E can be anti-inflammatory through inhibition of the PKC pathway (Lloret et al., 2019). It can activate protein phosphatase 2A (PP2A) that deactivates PKC and modulates diacylglycerol kinase (DGK) activity (Lloret et al., 2019). α- and β-tocopherol have different effects on PKC. α- tocopherol has a significant inhibitory effect on PKC in vascular smooth muscle causing arrest of cell growth but β-tocopherol does not (Betti et al., 2006; Lloret et al., 2019). This effect has been studied with regards to Alzheimer’s disease, as there is thought to be an oxidative stress and inflammatory component.

Vitamin E is thought to have anti-thrombotic properties and has been studied in myocardial infarction prevention. Separate from its antioxidant properties, α-tocopherol regulates genes and enzyme activity involved in vitamin E uptake and metabolism in addition to regulating lipoprotein uptake and inflammation (Ziegler et al., 2020). A study of patients with myocardial infarction, showed decreased levels of vitamin E in a significant number of patients (Ziegler et al., 2020). Anti-inflammatory properties including decreasing the release of proinflammatory cytokines including interleukin-8 (IL-8) and plasminogen activator inhibitor-1 (PAI-1) as well as decreasing CRP levels have been observed (Singh et al., 2005).

### Vascular Endothelial Growth Factor Signaling

Studies indicate vitamin E to have regulatory effects on angiogenesis through the modulation of VEGF (Zingg, 2019). The exact regulatory pathways are unclear, as in some settings vitamin E can activate or inhibit VEGF but it is thought to block effector mechanisms (Nussenblatt et al., 2006). Studies have been done in human microvascular endothelial cells (HMVECs), HUVECs, and cultured endothelial cells with varying effects on VEGF receptors (Zingg, 2019). It has been observed that expectant ewes given vitamin E showed enhanced angiogenesis and formation of the vascular network in the placenta, thought to be due to increased VEGF (Zingg, 2019). On the other hand, in a comparative study, tocotrienols inhibited bovine aortic endothelial cell proliferation and tube formation of which δ-tocotrienol appeared to have the highest activity. δ-tocotrienol reduced VEGF-activated tube formation in HUVECs and halted new blood vessel formation shown by a chorioallantoic membrane assay to assess *in vivo* angiogenesis on the growing chick embryo (Miyazawa et al., 2004).

## Molecular Mechanisms of Vitamin E Toxicity

Toxic amounts of vitamin E do not concentrate in the body as it is metabolized and transported out of the liver through bile and urine. This prevents accumulating α-tocopherol levels and eliminates toxic effects in most healthy individuals. Although rare, there are some circumstances where exogenous vitamin supplementation is not due to diet alone or conditions that prevent excess vitamin E from being eliminated from the body. Ideally, vitamin E supplementation should be kept to a lower dosage.

### Hypervitaminosis

As with many vitamins, an excess of vitamin E can cause potential health complications. Many individuals consume vitamin E supplements for its antioxidant or immune-boosting properties. An accumulation of vitamin is a pathological condition known as hypervitaminosis. It may take months for a vitamin to substantially accumulate in the tissues, especially if the body is unable to eliminate it (Kitagawa and Mino, 1989; Handelman et al., 1994). The daily tolerable upper limit dose is 1000 mg (Miller et al., 2005). An upsurge in mortality from all causes has been reported with excessive doses of vitamin E (Miller et al., 2005). Toxic amounts of vitamin E can alter liver and kidney function and cause muscle weakness or bleeding problems (Tsai et al., 1978). In addition, as vitamin E is metabolized in the liver by cytochrome P450 (CYP) enzyme system, it can interact with many other commonly used medications that share this pathway.

### Cardiovascular Disease

The link between high doses of vitamin E and heart failure has been extensively studied in several randomized control trials including most notably the HOPE and HOPE-TOO trials. These trials found greater risk of heart failure related to high doses of vitamin E (≥400 IU/d) (Lonn et al., 2005). The cause for this effect is unclear but has been postulated to be due to disruption of the natural balance of antioxidant systems or reduction of HDL cholesterol (Lonn et al., 2005). This effect was redemonstrated by a similar trial, finding a 50% increase in risk to develop congestive heart failure (CHF) after administration of α-tocopherol (Brigelius-Flohé, 2007). A study examining the effects of megadoses of vitamin E showed an increase in serum triglyceride levels most pronounced in female subjects (Tsai et al., 1978). Heart failure can result in microvascular dysfunction in the retina where dilation responses of both arteriolar and venular retinal microvessels were significantly reduced to a flicker stimulus (Grassi and Mancia, 2018; Nägele et al., 2018). Concern about this effect may limit the usefulness of vitamin E in large doses in patients with extensive cardiovascular risk factors or diabetes, both of which are common in the elderly population where AMD would be seen. On the other hand, Bursell et al. (1999) described in early stages of type I diabetes, high doses of vitamin E controlled retinal blood flow with minimal to no diabetic retinopathy.

### Thyroid Homeostasis

Tocopherol has been shown to affect the hypothalamus-pituitary-thyroid axis (Tsai et al., 1978). Prolonged vitamin E deficiency has shown a reduction in function of this system, while large doses of vitamin E have been shown to lower concentrations of thyroid hormone T3 and T4 (Tsai et al., 1978). However, studies with a longer duration have shown that this may be a transitory effect (Tsai et al., 1978). Low thyroid levels can cause hypertriglyceridemia and may contribute to this observed effect (Tsai et al., 1978). Thyroid hormones are known to regulate visual functions in human and mouse studies (Takeda et al., 1994, 1996; Ittermann et al., 2014). Data indicate that cultured human RPE cells are a direct target of thyroid hormones (THs; Duncan et al., 1999). Ma et al. (2014) looked at cone cell viability and whether TH signaling affects retinal degeneration mouse models. TH signaling has been shown to be important for cone visual pigment expression and pattern formation while an overabundance of TH signaling causes cone degeneration (Ng et al., 2010). In contrast, Ma’s study discovered when TH signaling was suppressed in rodent cone-rod dystrophy models, preservation of cones was found, a novel approach to macular degeneration therapy (Ma et al., 2014). A link between thyroid hormone, vitamin E, and macular degeneration has not been thoroughly investigated and can only be speculated at this time.

### Bleeding Disorders

The most well-known symptom of vitamin E toxicity is bleeding. Vitamin E inhibits vitamin K dependent activation of clotting factors, tissue factor, and inhibits aggregation of platelets with an oxidative stress mediated mechanism (Handelman et al., 1994; Chapy et al., 2015). Intracranial hemorrhagic stroke with higher than recommended doses of vitamin E has been reported (Le et al., 2020). This is especially important to consider in patients using warfarin (a vitamin K antagonist) for anticoagulation. A retrospective cohort study found that serum vitamin E levels could predict bleeding events in patients on warfarin (Chapy et al., 2015).

### Drug Interactions

Vitamin E supplementation can also potentially interact with medications such as simvastatin (Zocor) and niacin, chemotherapy and radiotherapy, and anticoagulants and antiplatelet medications (Brown et al., 2001; Cheung et al., 2001; Doyle et al., 2006; Block et al., 2007; Lawenda et al., 2008; Violi et al., 2010; Pastori et al., 2013). Decreased concentrations of mRNA for hepatic organic anion transporting polypeptide 3 (OATP3) transport proteins have been found in rats that were injected with α-tocopherol (Podszun and Frank, 2014). These transport proteins are important for uptake of statin medications into circulation. Several cases have implicated the use of niacin with cystoid macular edema (CME; Millay et al., 1988; Fraunfelder et al., 1995; Callanan et al., 1998; Domanico et al., 2015). The CYP enzyme family can also be affected by vitamin E. These enzymes are responsible for metabolism of xenobiotics and 60% of prescription medications (Brigelius-Flohé, 2007). In rat studies using vitamin E supplementation, a vitamin E deficient diet reduced CYP enzyme concentrations. The mechanism behind this is thought to be vitamin E activation of a nuclear pregnane X receptor (PXR) driven chloramphenicol acetyltransferase (CAT) reporter in HepG2 cells, a human hepatoma cell line that is typically used in drug metabolism and hepatotoxicity studies which can mediate and induce CYP functions (Brigelius-Flohé, 2007). This can decrease the efficacy of common drugs. RRR-α-tocopherol did not alter hepatic mRNA expression of CYP enzymes, however, high doses of all racemic α-tocopherol acetate induced hepatic mRNA expression 3-4x (Podszun and Frank, 2014). At normal doses, vitamin E does not appear to have significant effects on CYP expression. There is evidence to suggest a link between CYP27A1, a broadly expressed mitochondrial sterol 27-hydroxylase, AMD, and cholesterol maintenance in the retinal. Retinal lesions developed in Cyp27a1^–/–^ mice were characterized by cholesterol containing drusen, neovascularization, and activated Müller cells (Omarova et al., 2012). Müller cells are the first to reveal changes in metabolic processes due to retinal stress or disease. Cholesterol buildup is associated with macular degeneration (Sarks et al., 1999; Curcio et al., 2011). It is conceivable that the AREDS formulation containing vitamin E reduces the progression of drusen in AMD, but this is not proven and remains to be seen.

### Fetal Health and Birth Defects

Research investigating teratogenic effects triggered by vitamin E on fetal health has been investigated in rat models where no obvious teratogenic effects, survival rate, or size and weight of litters were observed (Martin and Hurley, 1977). In mothers treated daily with 500 mg of vitamin E, results showed a delay in opening eyelids and other ocular complications, however, nothing statistically significant in comparison to control animals (Martin and Hurley, 1977). It has been shown in several studies that vitamin E protects against birth defects in the presence of nicotine use, sparing malformations, embryonic bone development (Güler et al., 2022), decreased the rate of embryo malformations, and increased size and maturation of streptozotocin-induced diabetic animals (Viana et al., 1996). Folic acid and vitamin E taken together with antiepileptic, antihypertensive, and anti-allergic drugs prevented mortality and teratogenic effects in mice (Wahid et al., 2014). Teratogenicity due to zinc deficiency is not ameliorated by vitamin E, suggesting that fatty acid metabolism may be impeded by zinc causing an increase in the lipid peroxidation rate (Hurley et al., 1983). With this, it cannot be assumed that vitamin E’s antioxidant effects are entirely beneficial.

### Ocular Drug Delivery to Bypass Systemic Effects

While significant doses of systemic vitamin E have demonstrated a toxic effect (Abdo et al., 1986), there is less pre-clinical evidence to show that accumulations of ocular vitamin E are toxic. Intravitreal injection of high concentrations of an ocular therapeutic allows for the bypass of systemic effects (Peyman et al., 2009). Results from intravitreal injection of vitamin E in rabbits suggest that α-tocopherol in doses of 0.05, 0.10, and 0.20 mL failed to show any toxic effects following injection at 1 week, 1 month, and 3 months (Fallor et al., 1984).

## Conclusion and Future Research

Vitamin E influences cell physiology and survival by several signaling pathways. The molecular mechanisms by which it achieves uptake, transport, metabolism, and cellular action to promote neuroprotective effects in the retina are still being elucidated. Clinical studies suggest that supplements containing vitamin E may benefit individuals with moderate to severe AMD in contrast to only a nominal protective effect in early disease progression. Reducing the risk of AMD vision loss must begin prior to detection. Supplementing our diet with vitamin E, recommended by medical professionals, is beneficial to our health and survival but current literature also warns us against adverse effects. The long-term supplementation of vitamin E to counteract the progressive effects of AMD deserves further pre-clinical research.

## Author Contributions

PK conceived and designed the review. All authors performed the literature review, wrote, edited, and reviewed the manuscript.

## Conflict of Interest

The authors declare that the research was conducted in the absence of any commercial or financial relationships that could be construed as a potential conflict of interest.

## Publisher’s Note

All claims expressed in this article are solely those of the authors and do not necessarily represent those of their affiliated organizations, or those of the publisher, the editors and the reviewers. Any product that may be evaluated in this article, or claim that may be made by its manufacturer, is not guaranteed or endorsed by the publisher.
